# Hemostatic Proximal Anastomosis for Type A Acute Aortic Dissection: Reversed Turn-Up Technique (Akita Method)

**DOI:** 10.7759/cureus.74647

**Published:** 2024-11-28

**Authors:** Ryo Shimano, Takuya Komatsu, Junzo Inamura, Suguru Miyazaki, Masafumi Akita

**Affiliations:** 1 Cardiovascular Surgery, Shinmatsudo Central General Hospital, Chiba, JPN; 2 Cardiovascular Surgery, Kobari General Hospital, Chiba, JPN; 3 Cardiac Surgery, International University of Health and Welfare Mita Hospital, Tokyo, JPN

**Keywords:** ascending aorta replacement, hemostatic, proximal anastomosis, suture bleeding, turn-up technique, type a aortic dissection

## Abstract

In surgery for acute type A aortic dissection, controlling bleeding from the posterior wall of the proximal anastomosis is particularly challenging. To address this, we use the “reversed turn-up technique.” For the reinforcement of the proximal aortic stump, Teflon felt strips were placed inside and outside the suture line with 4-0 polypropylene continuous transverse mattress sutures, and BioGlue was applied to the false lumen. For the posterior wall, circumferential interrupted sutures using 3-0 polypropylene with pledgets were placed, passing the needle from the inside to the outside of the aorta and from the outside to the inside of the graft, resulting in an “inward” turn-up of the posterior wall. Continuous sutures were added for further reinforcement. For the anterior wall, circumferential interrupted sutures using 3-0 polypropylene with pledgets were placed, passing the needle from the outside to the inside of the aorta and from the inside to the outside of the graft, achieving an outward turn-up. This technique provides reliable hemostasis, particularly for the posterior wall. From August 2016 to January 2024, we performed initial and isolated ascending aortic replacement for acute type A aortic dissection in 73 patients using the reversed turn-up technique. The postoperative 30-day mortality rate was 4.1%, and no patients required re-exploration for bleeding.

## Introduction

In surgery for acute type A aortic dissection, achieving secure hemostasis at the anastomosis is critically important. Several effective anastomotic techniques have been reported [1-3]. Bleeding from the posterior wall of the proximal anastomosis, however, remains particularly challenging to control. To address this issue, we use the “reversed turn-up technique," which involves turning the proximal posterior wall inward. In this report, we describe our anastomosis technique and its outcomes.

## Technical report

When the entry tear is confined to the ascending aorta, ascending aortic replacement is our preferred procedure. The circulatory arrest is initiated at a tympanic temperature of 27°C, followed by selective antegrade cerebral perfusion through the three arch branches.

Distal anastomosis

**Figure 1:**
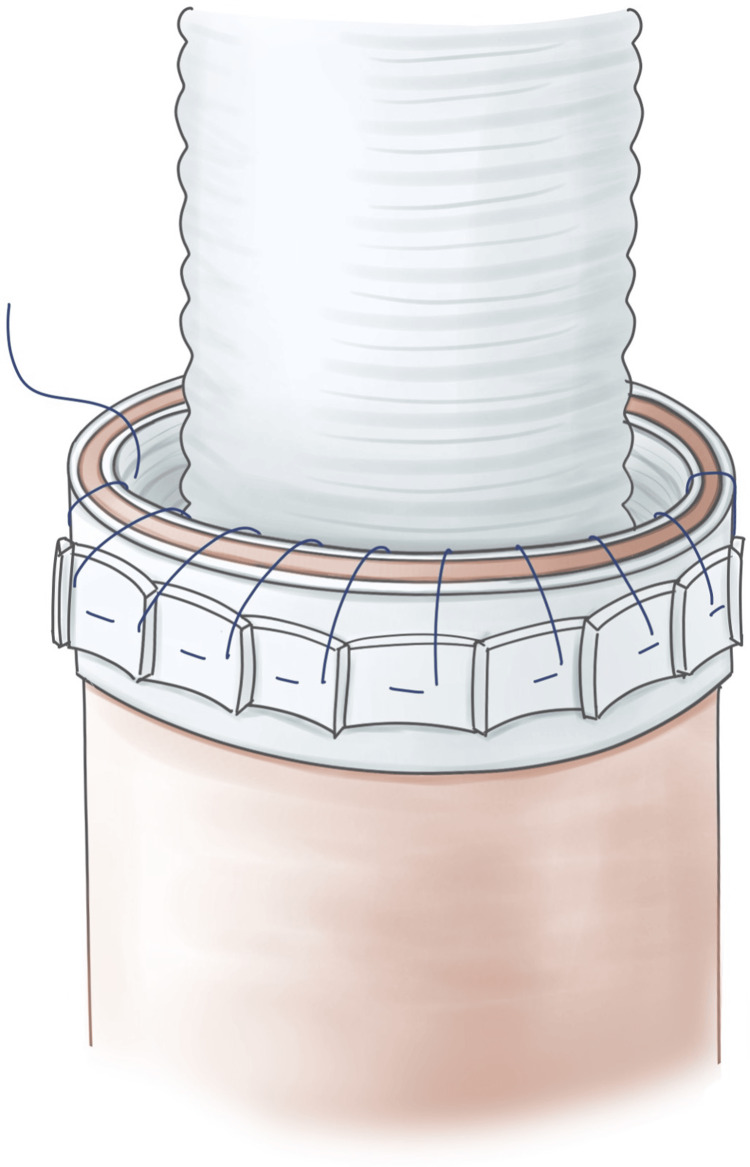
Schema of distal anastomosis. Teflon felt strips are placed inside and outside the suture line. After circumferential interrupted sutures are performed using 3-0 polypropylene with pledgets to evert the graft, additional circumferential 3-0 polypropylene continuous sutures are placed. Image Credits: Ryo Shimano

To reinforce the distal aortic stump, Teflon felt strips are placed inside and outside the suture line with 4-0 polypropylene continuous transverse mattress sutures, and BioGlue (CryoLife Inc., Kennesaw, GA, USA) is applied to the false lumen. Continuous circumferential interrupted sutures are performed using 3-0 polypropylene with pledgets, securing the graft in an everted fashion. Perfusion is resumed through the graft side branch, followed by additional 3-0 polypropylene continuous sutures and BioGlue application to the suture line (Figure 1).

Proximal anastomosis (reversed turn-up technique)

**Figure 2:**
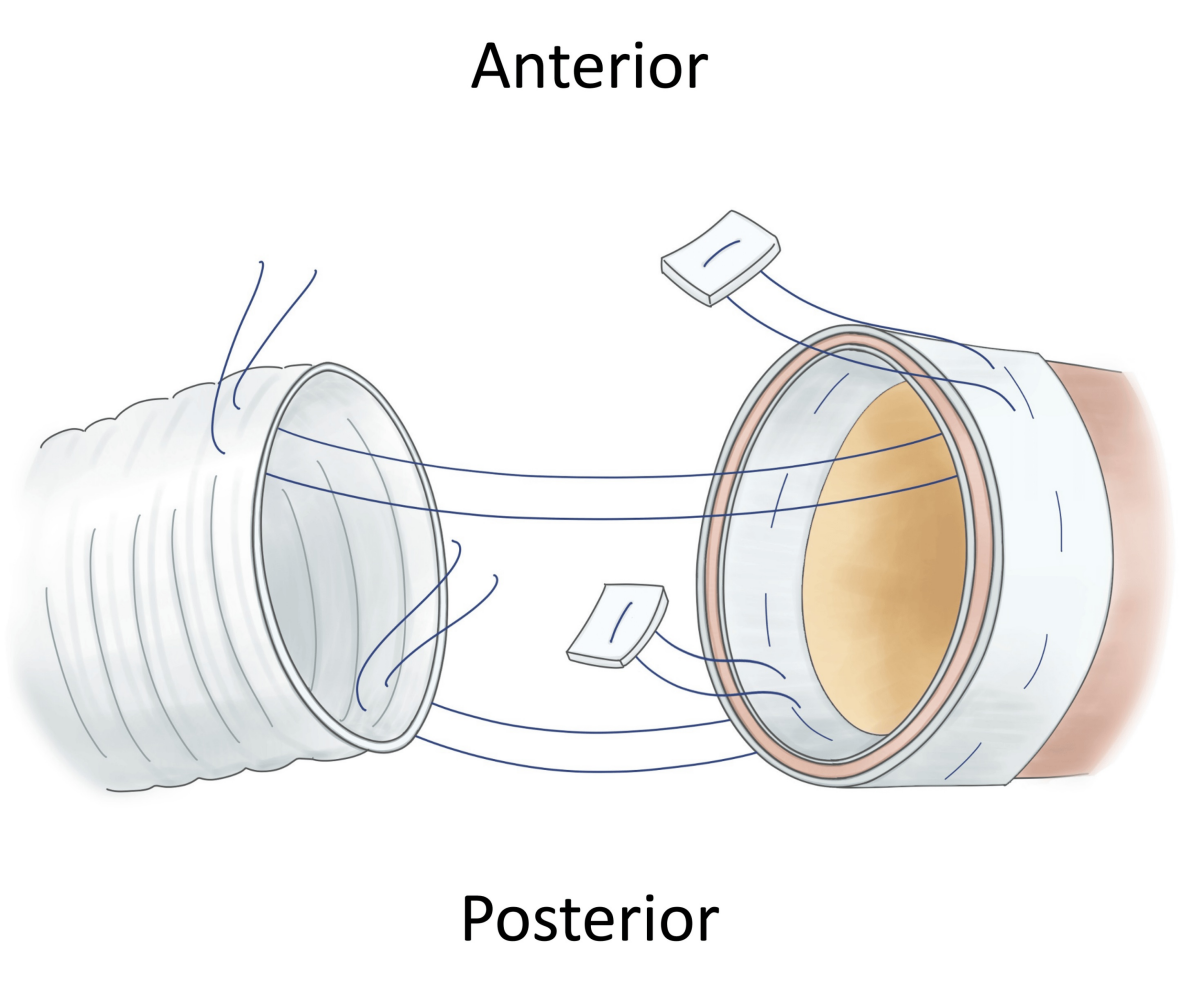
Conceptual image of the reversed turn-up technique for proximal anastomosis. For the posterior wall, the needle is passed from the inside to outside of the aorta and then from the outside to inside of the graft. By contrast, for the anterior wall, the needle is passed from outside to inside of the aorta and then from inside to outside of the graft. Image Credits: Ryo Shimano

**Figure 3:**
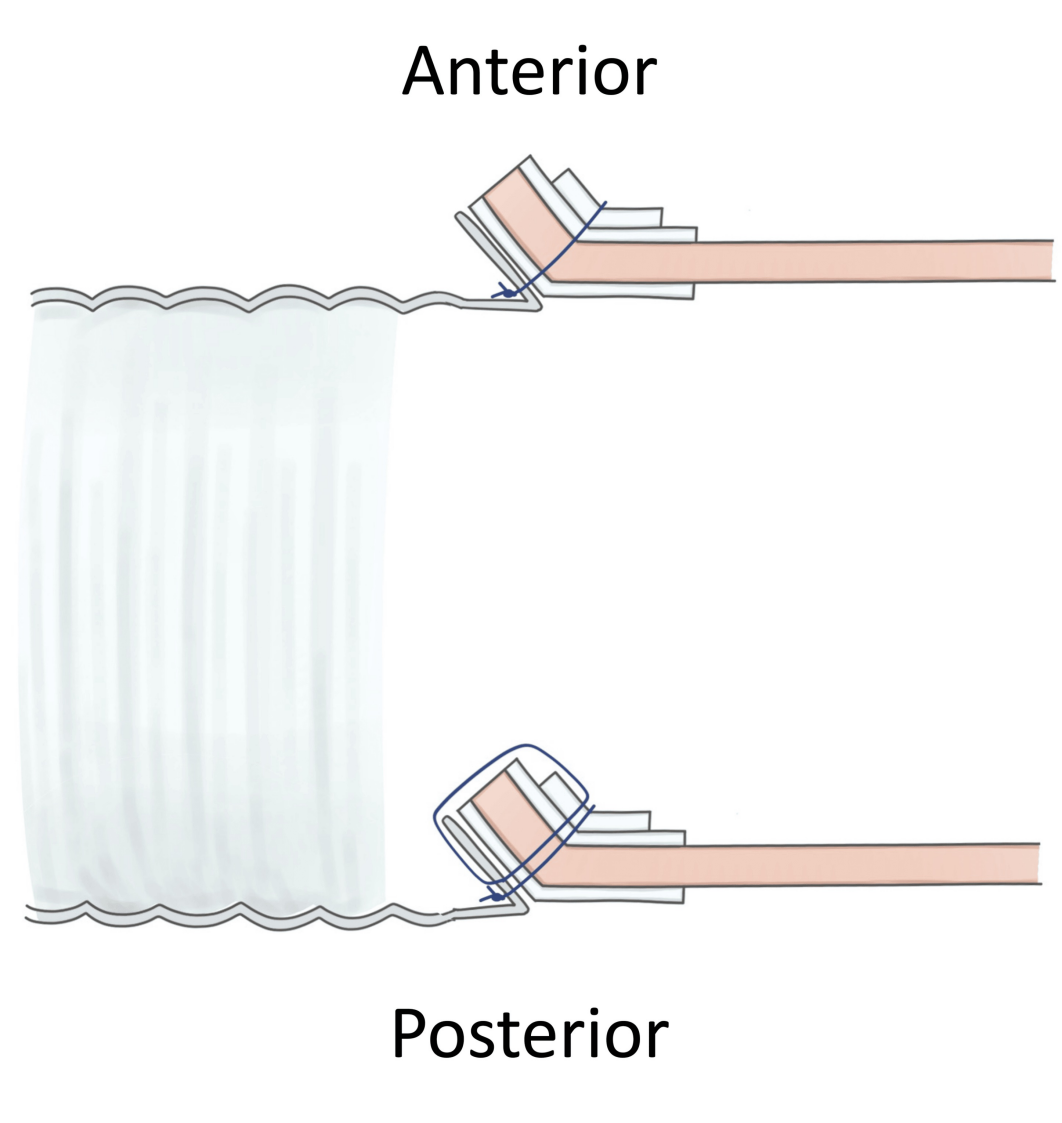
Cross-sectional schema of the reversed turn-up technique for proximal anastomosis. The posterior wall is turned up inward, while the anterior wall is turned up outward. Image Credits: Ryo Shimano

**Figure 4:**
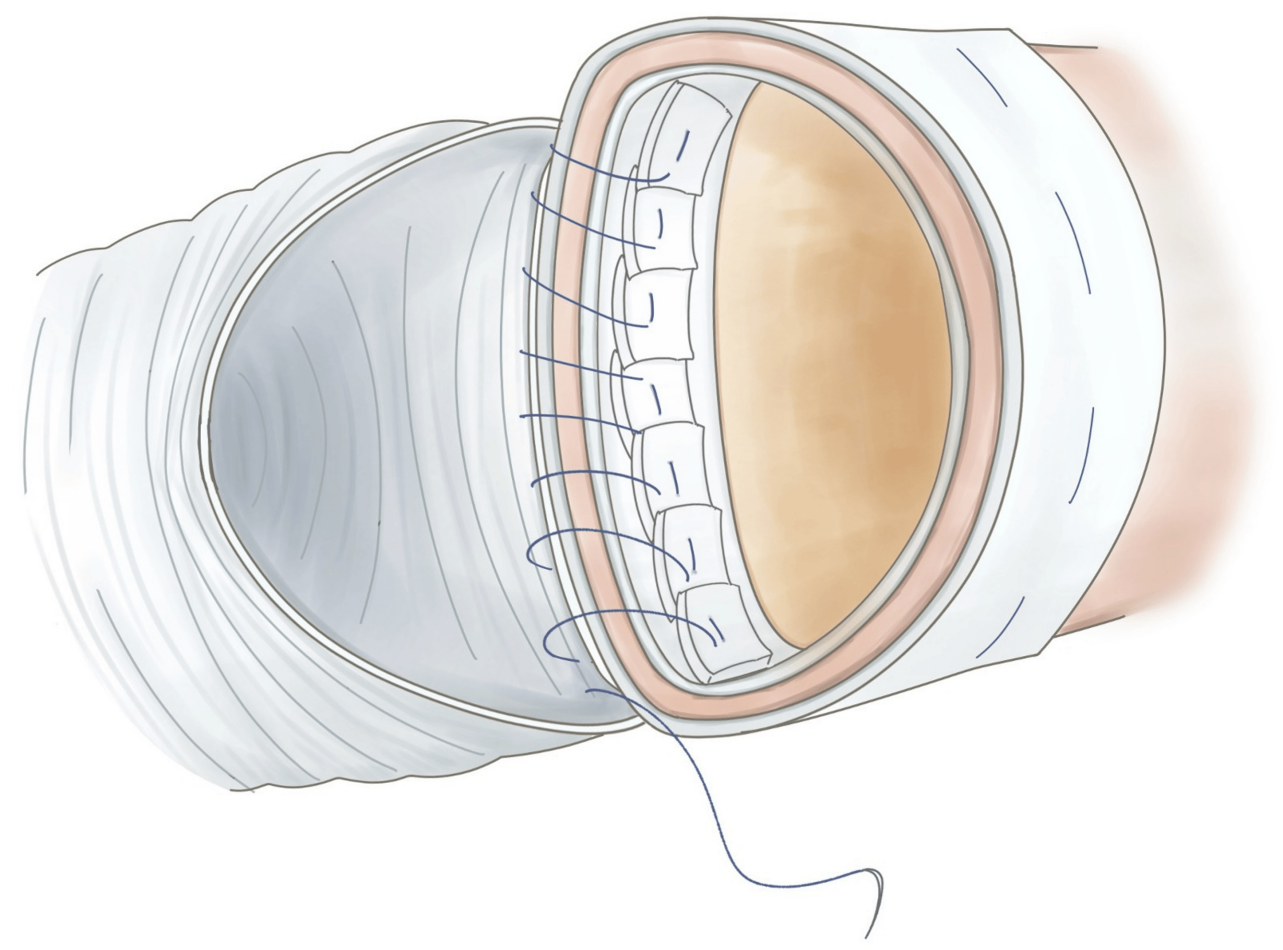
Schema showing additional continuous sutures on the proximal posterior wall. Image Credits: Ryo Shimano

Reinforcement of the proximal aortic stump is performed in the same manner as for the distal stump. For the posterior wall, circumferential interrupted sutures using 3-0 polypropylene with pledgets are placed by passing the needle from the inside to outside of the aorta and from the outside to inside of the graft, resulting in an “inward” turn-up of the posterior wall. 3-0 polypropylene continuous sutures are added for reinforcement. For the anterior wall, circumferential interrupted sutures using 3-0 polypropylene with pledgets are placed by passing the needle from outside to inside of the aorta and from inside to outside of the graft, creating an outward turn-up (Figures 2-4). Overlapping pledgets in the interrupted sutures further enhance hemostasis. The reversed turn-up technique, developed by co-author Masafumi Akita, is also referred to as the “Akita method.”

From August 2016 to January 2024, we performed initial and isolated ascending aortic replacement for acute type A aortic dissection in 73 patients using this anastomosis technique. Of these, 26 procedures were performed by early-career surgeons who were not board-certified as cardiovascular specialists. The results are shown in Table 1.

**Table 1:** Operative data SD, standard deviation; CPB, cardiopulmonary bypass; RBC, red blood cells; FFP, fresh frozen plasma The term “early-career surgeon” refers to a surgeon who has not yet obtained board certification as a cardiovascular specialist.

	n = 73
Age (years, mean ± SD)	72.5 ± 10.5
Male	29 (39.7%)
Operation time (min, mean ± SD)	271.3 ± 71.1
CPB time (min, mean ± SD)	182.1 ± 46.7
Cardiac arrest time (min, mean ± SD)	136.3 ± 39.6
Circulatory arrest time (min, mean ± SD)	55.7 ± 18.8
Intraoperative bleeding (ml, mean ± SD)	1017.5 ± 419.2
Intraoperative RBC (unit, mean ± SD)	10.4 ± 4.1
Intraoperative FFP (unit, mean ± SD)	14.1 ± 4.1
Early-career surgeons performed	26 (35.6%)
Postoperative drainage at 12 hours (ml, mean ± SD)	598.7± 586.7
30-day mortality	3 (4.1%)
Re-exploration for bleeding	0 (0%)
Stroke	7 (9.6%)
Mediastinitis	3 (4.1%)
Paraplegia	0 (0%)

## Discussion

Causes of anastomotic bleeding include gaps resulting from insufficient adhesion between the aorta and the vascular graft, as well as bleeding from needle holes, which is more common in cases of aortic dissection because of the fragility of the aorta [4]. In the reversed turn-up technique, the posterior wall is turned up inward, ensuring strong adhesion between the aorta and the graft even under high blood pressure. In addition, the needle holes on the posterior wall are located inside the vessel and remain unexposed, effectively preventing needle hole bleeding. The areas corresponding to 3 and 9 o’clock, which mark the boundaries between the inwardly and outwardly turned-up portions, may occasionally bleed. However, because these areas are located on the lateral wall, additional sutures for hemostasis can be easily placed. In theory, the inversion of the posterior wall could lead to the narrowing of the vessel lumen. This narrowing is mitigated by making the posterior wall bites relatively small (approximately 4-5 mm). Moreover, adding continuous sutures to the posterior wall allows the protruding portion inside the lumen to retract to some extent. We have not encountered any complications, such as hemolysis, that could be attributed to the narrowing of the vessel lumen.

According to a report from Japan [5], the 30-day mortality rate following ascending aorta replacement for acute type A dissection is 6.8%. Given that more than one-third of these surgeries at our institution were performed by early-career surgeons who were not yet board-certified as cardiovascular specialists, the reversed turn-up technique demonstrates that satisfactory surgical outcomes can be achieved regardless of the surgeon’s level of experience.

## Conclusions

The reversed turn-up technique offers reliable hemostasis and likely contributes to improved surgical outcomes. Its technical ease and simplicity make it an effective anastomotic method that can be utilized regardless of the surgeon’s experience. Further clinical validation and research are warranted to confirm its efficacy.

## References

[1] Tamura N, Komiya T, Sakaguchi G, Kobayashi T (2007). 'Turn-up' anastomotic technique for acute aortic dissection. Eur J Cardiothorac Surg.

[2] Inoue Y, Minatoya K, Itonaga T (2016). Utility of proximal stepwise technique for acute aortic dissection involving the aortic root. Ann Thorac Surg.

[3] Nakahara Y, Tateishi R, Haba F, Ono S, Kanemura T (2024). Insertion multi-parachute suturing and knotting (IMS-K) technique for aortic surgery. Cureus.

[4] Mori K, Kozaki S, Anai H, Wada T, Shuto T, Umeno T, Miyamoto S (2020). Gorget-like cuddling suture: an anastomosis reinforcement technique to reduce suture bleeding for aortic surgery. Ann Vasc Surg.

[5] Yoshimura N, Sato Y, Takeuchi H (2024). Thoracic and cardiovascular surgeries in Japan during 2021 : annual report by the Japanese Association for Thoracic Surgery. Gen Thorac Cardiovasc Surg.

